# Indiana Medical Protective Law

**Published:** 1879-07

**Authors:** 


### EDITORIAL, ETC.

*Indiana Medical Protective Law.*—A recent enactment of the legislature of Indiana provides that all legally chartered Medical Colleges in that state shall have appointed from the State Medical Association *of the same school of practice*, to which such College belongs, a "Board of Examiners," who shall examine all candidates for graduation, and shall recommend or not the bestowal of diplomas. Graduates five years in practice, and non-graduates two years in practice previous to the passage of this act, are exempt from its provisions. Each candidate for practice, not thus excepted, is required to present his diploma to the clerk of the court, before he can obtain license to practice. The penalty for violating this law is imprisonment in jail not less than six months, and $25 fine. In force from its passage.

Is it not time that something of this sort was done in dentistry, and could not a concerted move be made by the profession which would bring about the enactment of some such law? If the practice of medicine needs protection,—and that it does need it is evident from the above and similar moves in other states than Indiana,—surely we need protection as well, in our scope and range. The flood of candidates for graduation in the dental schools, and the strong pressure brought to bear on those having charge of these institutions, has its natural effect on even those whose intentions are the best; as a consequence a revision of the decisions of inside examining "Faculties" by outside and less sympathetic "Examining Boards," would doubtless check the tendencies which now exist, and which must exist so long as the relations of teacher and student are what they are. The subject is one of difficulty, and it is hard to see how the adoption of such a rule on the part of dental colleges, or the construction of such a law, could rid the case of all its embarassments. Still, it is probable that less hardship would result than at present, for no hardship of rejection can equal that of graduates, incompetency—a double injury, to the student, and to the public.

Cannot those who mould public opinion; cannot the American Dental Association, or the Southern Association, set on foot some scheme which will result in the definite solution of this problem so far as our profession is concerned. The colleges are what the profession make them, and are ready to respond to pressure from without, but—it is in vain to hope that they will do more than this; in vain to hope that they will anticipate public professional desire publicly expressed.

*Apropos* of this subject, we notice that in Alabama, where were recently enacted laws providing for a State Board of Censors of the Medical Association of that State, *six out of seven* of the applicants to this board for diplomas were rejected outright; yet these men had been practicing or pretending to practice medicine for years. What has been done in Alabama for the protection of medical practice can be done in Maryland and Virginia for the protection of dental practice, and should be. Certainly every man who is competent should be allowed to practice dentistry, or any other calling; but for the protection of the public, the profession, and in the interests of the colleges themselves, as many guards as possible should be thrown around the subject. The dental student and candidate for graduation is doubtless as well trained as is the student of any other profession for his calling, but if additional guards are put *outside of and independent of the colleges*, it will for obvious reasons surely make these more careful. H.

